# Supplementary material for: Atypical intrinsic neural timescale in autism
Source: eLife. 2019 Feb 5;8:e42256. doi: 10.7554/eLife.42256 (PMC6363380; doi:10.7554/eLife.42256)
Supplement: Supplementary file 1. — Supplementary table 1: Dataset for the reproducibility test. Supplementary table 2: Properties of the longitudinal dataset. [file elife-42256-supp1.docx]

**Supplementary File**

for “Atypical intrinsic neural timescale in autism”

by Watanabe, Rees, and Masuda.

**Supplementary Table 1**

Dataset for the reproducibility test

|  | Typically developing (TD) | Autism spectrum (ASD) | *P* value |
| --- | --- | --- | --- |
| **ETH Zürich** |  |  |  |
| Number of participants | 15 | 10 | – |
| Age (Mean ± SD) | 23.2±3.3 (18–29) | 21.5±2.7 (18.5–27.25) | 0.2 |
| Sex | Male | Male | – |
| Laterality | Right | Right | – |
|  |  |  |  |
| Full IQ | 114.7±9.2  (100–133) | 108.9±12.8  (82–123) | 0.21 |
| Verbal IQ | 115±12.1  (97–139) | 109.5±14.5  (85–129) | 0.23 |
| Performance IQ | 112.7±10.1  (96–133) | 107.0±11.9  (84–123) | 0.21 |
|  |  |  |  |
| ADOS Social | – | 6.7±1.3 (5–9) | – |
| ADOS Communication | – | 2.5±1.1 (1–5) | – |
| ADOS RRB | – | 0.5±0.97 (0–3) | – |
|  |  |  |  |
| Head motion (mm) | 1.2±0.8 (0.13–2.3) | 1.5±0.9 (0.15–2.4) | 0.62 |
|  |  |  |  |
| **Indiana University** |  |  |  |
| Number of participants | 10 | 9 | - |
| Age | 23.0±3.8  (18–28) | 21.5±5.4  (19–37) | 0.7 |
| Sex | Male | Male | - |
| Laterality | Right-handed | Right-handed |  |
|  |  |  |  |
| Full IQ | 115.4±14.2  (93–131) | 120.6±6.7  (109–129) | 0.4 |
| Verbal IQ | 115.7±13.4  (88–130) | 123.3±14.6  (98–138) | 0.3 |
| Performance IQ | 110.7±13.1  (90–125) | 111.8±7.8  (102–127) | 0.9 |
|  |  |  |  |
| ADOS score |  |  |  |
| Social | - | 5.7±1.2 (4–8) | - |
| Communication | - | 2.2±1.3 (1–5) | - |
| RRB | - | 1.7±1.0 (0–3) | - |
|  |  |  |  |
| Head motion (mm) | 1.2±0.5 (0.21–2.1) | 1.4±0.5 (0.25–2.4) | 0.4 |

**Supplementary Table 2**

Properties of the longitudinal dataset.

|  | Typically developing (TD) | Autism spectrum (ASD) | *P* value |
| --- | --- | --- | --- |
| Number of participants | 7 | 11 | - |
| Number of scanning | 14 | 22 | - |
| Age at the 1st scan | 12.2±1.1  (10.9–13.6) | 11.4±1.0  (9.9–13.4) | 0.11 |
| Age at the 2nd scan | 15.2±1.1  (13.5–16.7) | 14.2±1.1  (12.5–15.6) | 0.1 |
| Sex | Male | Male | - |
| Laterality | Right-handed | Right-handed |  |
|  |  |  |  |
| IQ at the 1st scan |  |  |  |
| Full IQ | 116.1±12.7  (90–128) | 102.0±19.4  (84–132) | 0.1 |
| Verbal IQ | 113.9±11.1  (92–127) | 99.6±15.1  (83–130) | 0.1 |
| Performance IQ | 114.7±12.3  (91–129) | 103.3±18.5  (91–129) | 0.14 |
|  |  |  |  |
|  |  |  |  |
| ADOS score (1st scan) |  |  |  |
| Social | - | 9.2±1.3  (7–11) | - |
| Communication | - | 3.5±1.6  (1–5) | - |
| RRB | - | 3.2±1.5  (2–7) | - |
|  |  |  |  |
| ADOS score (2nd scan) |  |  |  |
| Social | - | 10.7±2.4  (7–14) | - |
| Communication | - | No data | - |
| RRB | - | 3.3±1.4  (1–6) | - |
|  |  |  |  |
| Head motion (1st scan) | 1.4±0.8 (0.22–2.7) | 1.5±0.8 (0.22–2.9) | 0.3 |
| Head motion (2nd scan) | 1.5±0.7 (0.21–2.7) | 1.7±0.9 (0.23–2.9) | 0.2 |
